# 3D-Printed Poly(ε-Caprolactone)/Hydroxyapatite Scaffolds Modified with Alkaline Hydrolysis Enhance Osteogenesis In Vitro

**DOI:** 10.3390/polym13020257

**Published:** 2021-01-14

**Authors:** Sangbae Park, Jae Eun Kim, Jinsub Han, Seung Jeong, Jae Woon Lim, Myung Chul Lee, Hyunmok Son, Hong Bae Kim, Yun-Hoon Choung, Hoon Seonwoo, Jong Hoon Chung, Kyoung-Je Jang

**Affiliations:** 1 Department of Biosystems & Biomaterials Science and Engineering, Seoul National University, Seoul 08826, Korea; sb92park@snu.ac.kr (S.P.); jsw3055@snu.ac.kr (S.J.); jwlim1130@snu.ac.kr (J.W.L.); josephmyungchul@gmail.com (M.C.L.); shmking@snu.ac.kr (H.S.); hbkim@snu.ac.kr (H.B.K.); 2 Department of Biosystems Engineering, Seoul National University, Seoul 08826, Korea; je6740@snu.ac.kr (J.E.K.); rhineop@snu.ac.kr (J.H.); 3 BK21 Global Smart Farm Educational Research Center, Seoul National University, Seoul 08826, Korea; 4 Department of Otolaryngology, Ajou University School of Medicine, Suwon 16499, Korea; yhc@ajou.ac.kr; 5 Department of Industrial Machinery Engineering, College of Life Sciences and Natural Resources, Sunchon National University, Suncheon 57922, Korea; 6 Interdisciplinary Program in IT-Bio Convergence System, Sunchon National University, Suncheon 57922, Korea; 7 Research Institute of Agriculture and Life Sciences, Seoul National University, Seoul 08826, Korea; 8 Division of Agro-System Engineering, College of Agriculture and Life Science, Gyeongsang National University, Jinju 52828, Korea; 9 Institute of Agriculture & Life Science, Gyeongsang National University, Jinju 52828, Korea

**Keywords:** surface modification, alkaline hydrolysis, oxygen plasma, 3D printing, hydroxyapatite, 3D scaffold

## Abstract

The 3D-printed bioactive ceramic incorporated Poly(ε-caprolactone) (PCL) scaffolds show great promise as synthetic bone graft substitutes. However, 3D-printed scaffolds still lack adequate surface properties for cells to be attached to them. In this study, we modified the surface characteristics of 3D-printed poly(ε-caprolactone)/hydroxyapatite scaffolds using O2 plasma and sodium hydroxide. The surface property of the alkaline hydrolyzed and O2 plasma-treated PCL/HA scaffolds were evaluated using field-emission scanning microscopy (FE-SEM), Alizarin Red S (ARS) staining, and water contact angle analysis, respectively. The in vitro behavior of the scaffolds was investigated using human dental pulp-derived stem cells (hDPSCs). Cell proliferation of hDPSCs on the scaffolds was evaluated via immunocytochemistry (ICC) and water-soluble tetrazolium salt (WST-1) assay. Osteogenic differentiation of hDPSCs on the scaffolds was further investigated using ARS staining and Western blot analysis. The result of this study shows that alkaline treatment is beneficial for exposing hydroxyapatite particles embedded in the scaffolds compared to O2 plasma treatment, which promotes cell proliferation and differentiation of hDPSCs.

## 1. Introduction

The development of an ideal bone graft material with excellent bone regeneration ability still remains a challenge for engineers [1]. The physicochemical properties of an ideal bone graft material have been reported in several studies [2,3]. In particular, a 3D scaffold requires enough space within its internal structure for cells to penetrate and form tissue. It is known that cell infiltration and proliferation are enhanced when the pore size reaches a scale of several hundred micrometers [4]. Additionally, there are numerous reports of cell proliferation on scaffolds with pore sizes of 300 to 600 micrometers [5]. To achieve this, methods such as gas foaming, salt leaching, solvent casting, particle leaching, ultrasonication, lyophilization, and thermally induced phase separation have been used. However, these methods cannot precisely control the internal structure and require complicated processing steps [4,6]. Therefore, 3D printing based on additive manufacturing is attracting attention as a technology that can overcome these drawbacks [7]. The creation of pores and the interconnectivity between them are considered important factors in the successful fabrication of a 3D scaffold [7]; thus, it is important to design the internal architecture of the scaffolds as well as the appropriate external structure. Additionally, 3D printing technology based on an additive manufacturing process can design and fabricate the internal structure of the scaffolds. This can greatly contribute to the production of highly interconnected porous scaffolds [8]. In particular, fused deposition modeling (FDM) 3D printers that utilize thermoplastic polymers present advantages, such as low price, fast production speed, and versatility of printing materials, and they are frequently used for tissue engineering research [9]. Thermoplastic polymer materials that can be used in FDM printers include synthetic polymers such as polylactic acid (PLA) and poly(lactic-co-glycolic acid) (PLGA), in addition to poly(ε-caprolactone) (PCL), all of which are approved by the U.S. Food and Drug Administration (FDA) [10]. Polymer scaffolds fabricated with FDM 3D printers in several studies easily maintained their shape and possessed excellent pore interconnectivity to supply the nutrients and metabolites necessary for cell infiltration and cell metabolism [11,12,13].

Since the physicochemical properties of the scaffold are influenced by the characteristics of the material itself, and a single type of material is used to produce the scaffold, various modification methods are used to overcome the drawbacks inherent to the component material [14,15,16]. As an example, PCL, approved by the FDA as an implanting material, exhibits the disadvantage that cell adhesion and interaction between cells are reduced due to the strong hydrophobicity of the surface; however, its mechanical strength and processability are favorable. Therefore, polymers with sufficient cell adhesion can be blended with a calcium apatite-based ceramic powder, which possesses strong cell affinity to maintain the mechanical strength of the scaffolds and increase the surface hydrophilicity and cell adhesion [10]. Many approaches have been reported to improve cell function. In particular, hydroxyapatite (HA) and β-tricalcium phosphate are indispensable elements for bone tissue regeneration and are known as materials that promote bone cell adhesion and osteoconduction and osteoinduction [17,18,19]. Due to these advantages, they have become popular as bone grafting materials in clinical practice.

In addition to blending polymers to provide cell attachment sites, the surface physical properties of PCL scaffolds can be changed by surface coating, plasma etching, alkaline hydrolysis, laser-induced grafting, and laser treatment, among other methods [10,20,21,22]. Plasma surface treatment is widely used in applications to improve the hydrophilicity of the material surface or create binding sites to which functional molecules can be attached [23]. The advantage of plasma treatment is that the chemical properties and morphology of the surface can be controlled with high precision without affecting the bulk physical properties of the material. In addition, plasma surface treatment does not use additional chemicals, and thus does not require additional steps such as washing; it also avoids the cytotoxic effect resulting from residual hazardous chemicals. Plasma treatment is widely used in biomedical applications because it can be modified to surfaces with different properties that arise from the chemical structure of the monomer of the polymer [24,25]. The technique exhibits high efficiency on 2D substrates; however, it has considerable limitations in 3D scaffolds because it cannot treat the internal region [26]. Alkaline hydrolysis is a method that involves a nucleophilic attack on the carbonyl carbon of ester and amide bonds and has been mainly used to increase the surface roughness and hydrophilicity of the material surface. In the hydrolysis reaction, not only are functional groups added to the surface of the polymer, but the polymer is also decomposed to form voids in the structure. According to previous reports, alkaline hydrolysis improves cell attachment and proliferation, and alkaline hydrolysis-treated PCL-tricalciumphosphate (TCP) scaffolds have shown increased bone formation compared to an untreated group [27]. Although hydrolysis has drawbacks such as decreasing the molecular weight of the polymer and diminishing its mechanical strength, advantages have also been reported, such as improving cellular behavior, facilitating osteogenesis, and controlling the degradation rate. In addition, the method can be applied to 3D scaffolds because it provides uniform treatment to the entire scaffold.

Human dental pulp-derived stem cells (hDPSCs) can be obtained from extracted teeth during surgery and harvested in large amounts while producing minimal tissue site morbidity [28]. Additionally, their rapid proliferation rate and interactivity with biomaterials make them more attractive for clinical applications [29]. In addition, hDPSCs are undifferentiated cells and have the ability to differentiate into multiple cell types under specific culture conditions including osteogenic, odontoblastic, neurogenic, and adipocytic cell lineages. Thus, hDPSCs are considered an important source of cells for bone tissue regeneration due to their distinct capacity to differentiate into osteoblasts [30].

**Figure 1:**
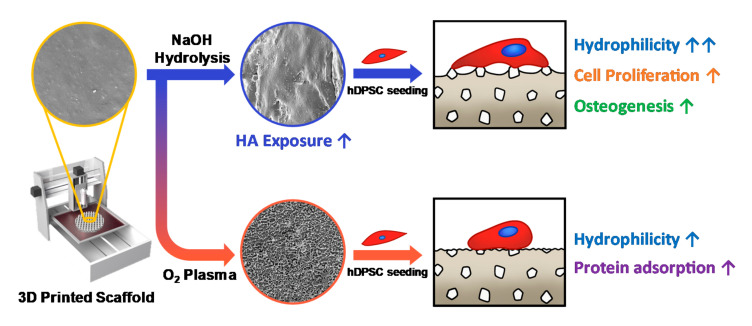
The schematic illustration of this study. We fabricated 3D scaffolds with different characteristics using a 3D printing process and surface modification techniques. NaOH treatment is a suitable method to expose the hydroxyapatite (HA) particles on the surface of the poly(ε-caprolactone) (PCL)/HA scaffold, which promotes proliferation and osteogenesis of human dental pulp-derived stem cells (hDPSCs).

In this study, we observed the effects of surface modification techniques on a 3D scaffold fabricated using PCL blended with HA powder on the growth and osteogenic differentiation of hDPSCs. We hypothesized that the HA powder in the PCL scaffold could not directly contact the cells due to the presence of PCL, so the surface was treated with O_2_ plasma, and the sodium hydroxide (NaOH) hydrolysis method was used to etch the surface of PCL (Figure 1). To evaluate the effects of the two methods on the properties of the materials, we measured the water contact angles, performed protein adsorption assays, and observed surface morphology via field emission-scanning electron microscopy (FE-SEM). Furthermore, the capacity of osteogenic differentiation was investigated through in vitro cell culturing on the 3D-printed scaffolds.

## 2. Materials and Methods

### 2.1. Preparation of 3D-Printed Scaffolds

In this experiment, PCL (MW: 45 000, Polysciences, Warrington, PA, USA), HA (Sigma-Aldrich, St. Louis, MO, USA) and dichloromethane (DCM; Duksan Co. Ltd., Ansan, Korea) were used. PCL films containing HA powders were fabricated using solvent evaporation. The PCL polymer was dissolved in DCM at a concentration of 20 wt%. HA powder was dispersed in ethanol at 5 wt% via ultrasonication for 5 min with an energy of 60 W. The HA powder-dispersed ethanol solution and the DCM solution containing the PCL polymer were blended at a volume ratio of 5:2 and stirred on a magnetic stirring plate at 500 rpm for 30 min. The well-mixed solution was poured into a square plate and the solvent was evaporated in a fume hood to obtain a PCL/HA mixture in film form. Next, approximately 10–20 g of the mixture was chopped, placed in a syringe, and loaded into the dispensing unit of the custom-made 3D printer. The material in the syringe was loaded into the barrel to allow it to preheat for 30 min at 85 °C to allow sufficient melting. Between 0.2 and 1 mL of DCM was added to the material and preheated for an additional 10 min. The 3D printing process was performed at 0.2 MPa and 1 mm/s.

### 2.2. Surface Modification of 3D-Printed Scaffolds

O_2_ plasma treatment and NaOH alkaline treatment were performed to modify the scaffold surface. O_2_ plasma treatment was carried out for 30 min at 2500 cc/min using CUTE (Femto Science, Hwaseong, Korea). NaOH (Duksan Co. Ltd., Ansan, Korea) solution of 10 M concentration was prepared, and each scaffold was immersed in the solution for 1 h. All samples were rinsed 5 times or more with deionized water to ensure no solvent remained.

### 2.3. Characteristics of 3D-Printed Scaffolds

The surface morphologies of the 3D-printed scaffolds were observed using field-emission scanning electron microscopy (FE-SEM; SUPRA 55VP, Carl Zeiss, Oberkochen, Germany). The scaffolds were sputter-coated with platinum and observed at an accelerating voltage of 2 kV. Energy-dispersive X-ray spectroscopy (EDS) was used to characterize the existence of HA particles in the scaffolds. In the FE-SEM image, strut and pore dimensions of the scaffolds were further measured using ImageJ software. To quantify the exposure of HA on the surface of the scaffolds, Alizarin Red S staining was performed. The scaffolds were stained with a 2% Alizarin Red S solution (Sigma-Aldrich, St. Louis, MO, USA) for 30 min at room temperature, followed by washing with deionized water. The scaffolds stained with Alizarin Red S were de-stained with a 10% cetylpyridinium chloride/10 mM sodium phosphate solution and incubated at room temperature for 30 min. The absorbance of the extracted stain was then measured using a microplate reader (Tecan, Männedorf, Switzerland) at 570 nm. In order to determine the content of HA particles in PCL/HA scaffold, thermogravimetric analysis (TGA; Q5000, TA instruments, New Castle, DE, USA) was performed. Samples were heated from room temperature to 800 °C at a heating rate of 10 °C/min. Mechanical properties of the scaffolds were measured according to ASTM D638 using a texture analyzer (TA.XT; Stable Micro Systems, Surrey, UK). Tensile strength, elastic modulus, and rupture strain of the scaffolds were analyzed. All the measurements were repeated five times on each scaffold. The hydrophilicities of the scaffolds were measured using a water contact angle analyzer (EasyDrop; Kruss, Hamburg, Germany). Briefly, droplets of 10 μL of water were dropped onto the surface of scaffolds, and the contact angle was recorded right after the contact. All the measurements were repeated five times on each scaffold.

### 2.4. Cell Attachment and Proliferation on the 3D-Printed Scaffolds

hDPSCs were obtained from a patient’s tooth at the Dental Hospital of Seoul National University (IRB: CRI05004). The hDPSCs were resuspended in a proliferating conditioned medium, alpha minimum essential media (α-MEM; Welgene, Gyeongsan, Korea), with a 10% fetal bovine serum (FBS; Welgene, Gyeongsan, Korea) and a 1% antibiotic antimycotic solution (Welgene, Gyeongsan, Korea). The hDPSCs were routinely maintained in a 5% CO_2_ incubator at 37 °C. To observe the cell attachment of the hDPSCs on the 3D scaffolds at 3 days of culturing, the adhered cells on the scaffolds were fixed with a 4% paraformaldehyde solution (Sigma-Aldrich, St. Louis, MO, USA) for 30 min, treated with 0.2% Triton X-100 (Sigma-Aldrich, St. Louis, MO, USA) for 15 min, and then stained with TRITC-conjugated phalloidin (Millipore, Burlington, MA, USA) for 1 h and 4, 6-diamidino-2- phenylindole (DAPI; Millipore, Burlington, MA, USA) for 5 min. A fluorescence microscope (Nikon, Tokyo, Japan) was used for acquiring the images of the stained cells. In order to evaluate the effect of the HA powder incorporation and scaffold surface modifications on the hDPSCs, the proliferation of hDPSCs on the various scaffolds was assessed via a WST-1 assay (Daeillab, Seoul, Korea). Briefly, the hDPSCs were seeded onto the scaffolds with 2 × 10^4^ cells/well. Cell proliferation was measured with the WST-1 assay kit on days 1, 3 and 7.

### 2.5. Osteogenic Differentiation of hDPSCs on 3D Scaffolds

The osteogenic differentiation capability of the hDPSCs on the scaffolds was assessed using Alizarin Red S staining. First, the hDPSCs were seeded onto the scaffolds with 8 × 104 cells/well and incubated for 24 h in a humidified CO2 incubator. Subsequently, the hDPSCs were substituted with osteogenic differentiation conditioned media, which consisted of α-MEM supplemented with 10% FBS, 1% penicillin, 0.1 μM dexamethasone (Sigma-Aldrich, St. Louis, MO, USA), 10mM B-glycerophosphate (Sigma-Aldrich, St. Louis, MO, USA), and 100μM ascorbic acid (Sigma-Aldrich, St. Louis, MO, USA). On days 10 and 20 of differentiation, the cells were rinsed with phosphate-buffered saline (PBS; Welgene, Gyeongsan, Korea) and fixed with 4% paraformaldehyde for 30 min. The fixed cells were treated with a 2% Alizarin Red R solution for 30 min at room temperature, followed by washing with deionized water. The stained cells were de-stained with a 10% cetylpyridinium chloride/10 mM sodium phosphate solution and incubated at room temperature for 30 min. Afterwards, the absorbances of the de-stained solution were measured using a microplate reader at 570 nm. To investigate the expression of proteins related to osteogenic differentiation, a Western blot assay was conducted after 10 and 20 days of differentiation. Briefly, osteogenic differentiation conditioned media were removed, and each sample was washed with PBS. After washing, cells were harvested from the scaffolds and treated with a cell lysis buffer (Millipore, Burlington, MA, USA), followed by incubation at 4 °C for 20 min and centrifugation at 13,000 rpm. The supernatant was collected and separated by 8% sodium dodecyl sulfate-polyacrylamide gel (SDS-PAGE) under reducing conditions. The protein was transferred to a polyvinylidene fluoride (PVDF) membrane (Millipore, Burlington, MA, USA) at 30 V for 1 h. The expression of osteopontin (OPN), osteocalcin (OCN), and runt-related transcription factor 2 (RUNX2) were observed. All the data were quantitatively analyzed using Image J software (NIH, Bethesda, MD, USA).

## 3. Results and Discussion

### 3.1. Characteristics of 3D-Printed Scaffolds

**Figure 2:**
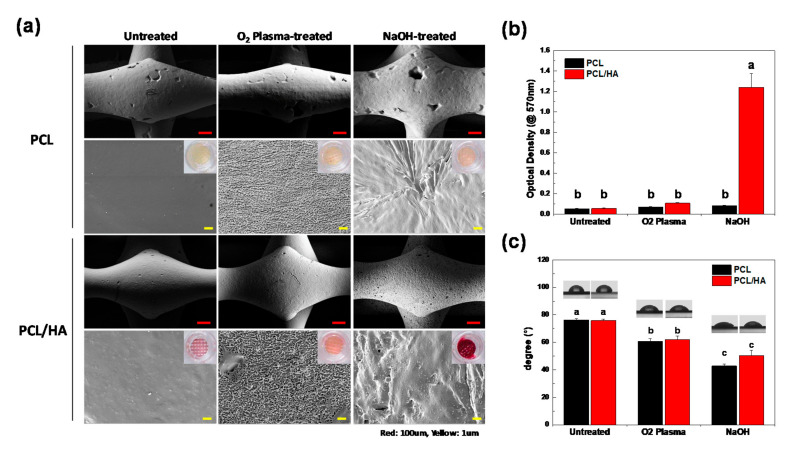
(**a**) FE-SEM image of the PCL and PCL/HA scaffolds before and after surface modification. Alizarin Red S staining was used to visualize the existence of hydroxyapatite on the scaffolds. (**b**) Quantitative data from the Alizarin Red S staining of the scaffolds (n = 3, ANOVA, Duncan’s multiple range test, *p* < 0.05). Same letters indicate that there is no significant difference between samples. (**c**) Water contact angle analysis of the scaffolds (n = 5, ANOVA, Duncan’s multiple range test, *p* < 0.05). Same letters indicate that there is no significant difference between samples.

The morphology of the 3D-printed PCL and PCL/HA scaffolds with different surface treatments was observed using FE-SEM (SUPRA 55VP, Carl Zeiss, Germany; Figure 2a). The non-treated PCL and PCL/HA scaffolds showed smooth surfaces, while the scaffolds with O_2_ plasma and NaOH treatments showed rough surfaces. This was an expected result for scaffolds subjected to O_2_ plasma and NaOH treatments [26,31]. The surface of the O_2_ plasma-treated scaffolds was finely etched, whereas that of the NaOH-treated scaffolds showed a relatively bulky surface morphology. The HA particles were uniformly distributed in the untreated PCL/HA scaffold but were not directly exposed on the surface because they were buried in the PCL. In the PCL/HA scaffold treated with NaOH, the internal HA particles were directly exposed on the surface. However, in the O_2_ plasma-treated PCL/HA scaffold, HA particles could not be observed on the surface because the O_2_ plasma reacted not only with the PCL but also with the HA particles, which were as a result volatilized. The existence of HA particles in the scaffolds was identified using EDS analysis (Supplementary Materials Figure S1 and Table S1). The EDS results confirmed that PCL/HA scaffolds contain the calcium ion which is a major element of HA. The pore and strut dimensions were further measured using FE-SEM images. The strut/pore dimensions of untreated, O_2_ plasma-treated, and NaOH-treated PCL scaffolds showed values of 265.7 μm/0.21 mm^2^, 260.7 μm/0.22 mm^2^, and 276.1 μm/0.20 mm^2^, respectively. The strut/pore dimensions of untreated, O_2_ plasma-treated, and NaOH-treated PCL/HA scaffolds showed values of 267.7 μm/0.23 mm^2^, 238.7 μm/0.25 mm^2^, and 256.8 μm/0.22 mm^2^, respectively (Figure S2). The strut dimensions showed a decreasing tendency due to the inclusion of HA particles. The reduced strut dimension caused an increase in the pore size.

In order to quantitatively analyze the existence of HA particles on the surface of the scaffolds [32], Alizarin Red S staining, which can detect a calcified matrix, was performed (Figure 2a). The scaffolds stained with Alizarin Red S were de-stained with a 10% cetylpyridinium chloride/10 mM sodium phosphate solution and incubated at room temperature for 30 min. The absorbance of the extracted stain was then measured using a microplate reader (TECAN, Switzerland) at 570 nm (Figure 2b). The PCL/HA scaffold with the NaOH treatment showed the highest de-staining level of Alizarin Red S compared to the other samples, confirming that the HA particles were exposed on the surface of the scaffold. The content of HA particles in the PCL/HA scaffold was investigated via TGA analysis. The weight loss of PCL/HA scaffolds was determined to be approximately 90% in the range 500–700 °C, thus ensuring that HA content to be 10%. The stress–strain curve for various scaffolds under tensile force is shown in Figure S4. Elastic modulus, tensile strength, and strain to rupture point were calculated and are presented in Table S2. The incorporation of HA particles did not cause a significant effect on elastic modulus and tensile strength of the scaffold, whereas it was observed that the PCL/HA scaffolds were much more brittle than the PCL scaffolds. Interestingly, NaOH treatment greatly increased the tensile strain of the scaffold, which can compensate for the decrease in mechanical properties due to the addition of HA particles. In addition, the hydrophilicity of the scaffolds was analyzed via water contact angle analysis (Figure 2c). The non-treated PCL and PCL/HA showed values of 76.3° and 75.8°, respectively. The water contact angles after O_2_ plasma treatment decreased to 60.7° and 62.2°, respectively, and after NaOH treatment, they decreased to 42.8° and 50.2°, respectively. Both the O_2_ plasma and NaOH treatments significantly increased the hydrophilicity of the scaffold surface.

### 3.2. Cell Attachment and Proliferation on the 3D-Printed Scaffolds

**Figure 3:**
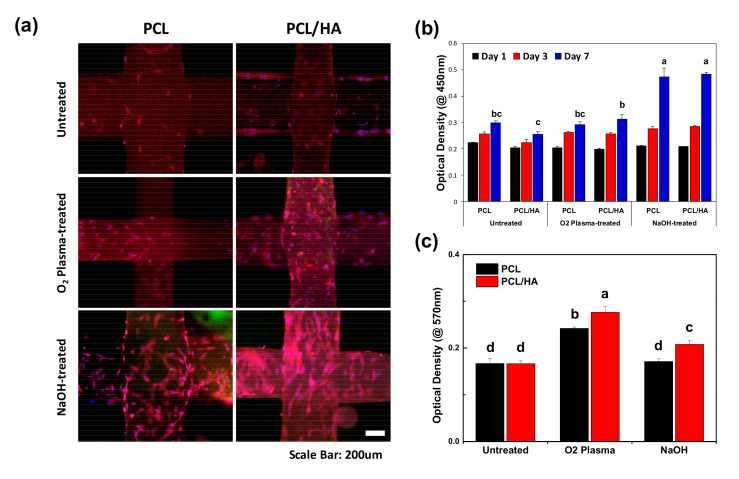
In vitro study of hDPSCs on the scaffolds. (**a**) Fluorescence microscopy image of the hDPSCs attached on the scaffolds on day 3. Both O_2_ plasma and NaOH treatments enhanced cell attachment of the scaffolds. (**b**) Cell proliferation on days 1, 3, and 7 using a WST-1 assay (n = 7, ANOVA, Duncan’s multiple range test, *p* < 0.05). At day 7, the NaOH-treated scaffolds exhibited significantly enhanced cell proliferation. Same letters indicate that there is no significant difference between samples. (**c**) Measurement of protein adsorption on the scaffolds after 24 h of incubation with 1% bovine serum albumin (BSA) solution (n = 5, ANOVA, Duncan’s multiple range test, *p* < 0.05). O_2_ plasma treatment greatly promoted protein adsorption ability of the scaffolds. Same letters indicate that there is no significant difference between samples.

In order to evaluate the biological effects of the surface modification techniques on the 3D-printed scaffolds, we conducted an in vitro study to investigate the cell attachment, proliferation, and differentiation of hDPSCs on the scaffolds. From the fluorescence microscopy images (Figure 3a), the hDPSCs were successfully attached and proliferated on the scaffolds after culturing for 3 d. The hDPSCs on the NaOH-treated scaffold were densely attached and showed an elongated morphology compared to those attached on the untreated or O_2_ plasma-treated scaffolds. The cell proliferation on the scaffolds was quantitatively analyzed by a WST-1 assay after culturing for 1, 3, and 7 d. On day 7, the proliferation of the hDPSCs cultured on the NaOH-treated PCL and PCL/HA scaffolds increased significantly compared to those cultured on the untreated and O_2_ plasma-treated samples. We found that the O_2_ plasma treatment greatly increased the protein adsorption ability of the scaffold which is closely related to cell adhesion [33]. The proliferation of hDPSCs could be influenced greatly by the hydrophilicity of the scaffold.

### 3.3. Osteogenic Differentiation of hDPSCs on 3D Scaffolds

**Figure 4:**
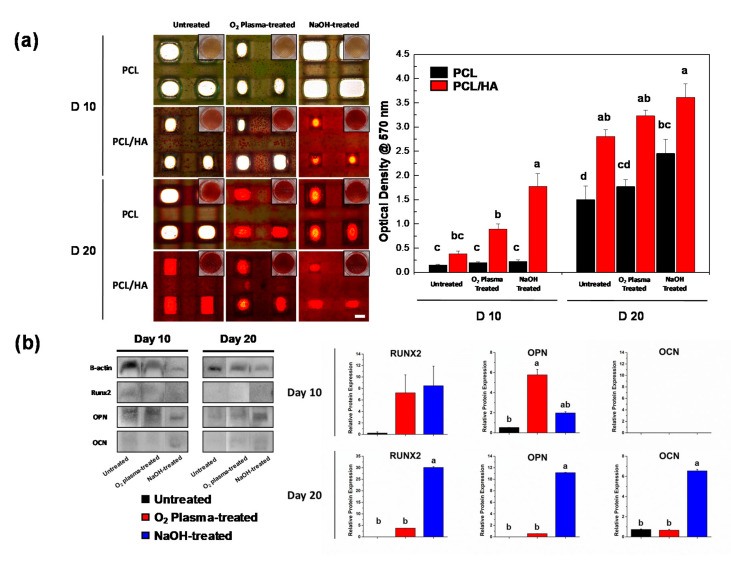
Osteogenic differentiation of hDPSCs on the scaffolds (**a**) Alizarin Red S staining of the hDPSCs cultured on the scaffolds on days 10 and 20. The quantitative data was obtained using de-staining (scale bar: 500 μm). The NaOH-treated PCL/HA group exhibited enhanced calcium deposition. Same letters indicate that there is no significant difference between samples. (**b**) The expression of osteogenic proteins on the PCL/HA scaffolds (RUNX2, OPN, and OCN) was determined by Western blotting on days 10 and 20. Quantitative results showed significantly enhanced RUNX2, OPN, and OCN expression on the NaOH-treated PCL/HA scaffold. (n = 3, ANOVA, Duncan’s multiple range test, *p* < 0.05). Same letters indicate that there is no significant difference between samples.

The osteogenic differentiation of the hDPSCs on various scaffolds was assessed by the Alizarin Red S staining and the Western blot assay on days 10 and 20 (Figure 4a). Generally, the Alizarin Red S staining method is used to detect the calcified matrix resulting from osteogenic differentiation. Here, the Alizarin Red S stain was further analyzed quantitatively to compare the matrix mineralization. The PCL/HA scaffolds treated with NaOH showed the highest bone mineralization among the samples. The HA particles exposed on the surface provided a bone-like microenvironment that promoted osteogenic differentiation of hDPSCs and consequently increased mineralization [34]. To further identify the enhanced osteogenic differentiation ability of the hDPSCs on the NaOH-treated PCL/HA scaffolds, protein expression of osteogenic differentiation markers was evaluated using a Western blot analysis (Figure 4b). On day 10, expression levels of runt-related transcription factor 2 (RUNX2) on the NaOH-treated PCL/HA were higher compared to untreated and O_2_ plasma-treated PCL/HA. RUNX2 is known as a modulator of early osteogenic differentiation in osteoblasts. RUNX2 binds with the cis-acting element of the osteocalcin (OCN) promoter region to trigger OCN expression [35]. On day 20, the expression levels of RUNX2, osteopontin (OPN), and OCN were highest in the NaOH-treated PCL/HA scaffold. An increased expression of osteogenic differentiation-related proteins indicated the ability of hDPSCs to differentiate into osteoblast cells [36]. These results illustrate that the HA particles exposed by the NaOH treatment played an important role in the osteogenic differentiation of hDPSCs.

## 4. Conclusions

Some 3D composite scaffolds with different surface characteristics were successfully fabricated using 3D printing and surface modification techniques. We investigated the effects of O_2_ plasma and NaOH treatment on the PCL/HA scaffolds to promote the surface and biological characteristics of the scaffold. After NaOH treatment, the hydrophilicity and protein adsorption ability of the PCL/HA scaffold were enhanced, which contributed to the adhesion and proliferation of hDPSCs. Additionally, NaOH treatment is a suitable method to expose the HA particles on the surface, which promotes osteogenic differentiation of hDPSCs. Therefore, NaOH treatment is an effective method for modulating the surface characteristics of the 3D-printed PCL/ceramic composite scaffolds for biomedical applications.

## Data Availability

All the experimental data herein presented are available on request from the corresponding author.

## Supplementary Materials

The following are available online at https://www.mdpi.com/2073-4360/13/2/257/s1, Figure S1: The EDS spectra of the PCL and PCL/HA scaffolds with different surface treatments, Figure S2: Strut and pore dimensions obtained for the different scaffolds, Figure S3: TGA curves of PCL and PCL/HA scaffolds, Figure S4: Stress-strain curves of PCL and PCL/HA scaffolds with different surface treatments. (×: Rupture Point, Black: untreated, Red: O_2_ plasma-treated, and Blue: NaOH-treated), Table S1: Elemental analysis of the scaffolds using EDS, Table S2: Mechanical properties of the scaffolds with different surface treatments.
