# Supplementary material for: 3D-Printed Poly(ε-Caprolactone)/Hydroxyapatite Scaffolds Modified with Alkaline Hydrolysis Enhance Osteogenesis In Vitro
Source: Polymers (Basel). 2021 Jan 14;13(2):257. doi: 10.3390/polym13020257 (PMC7830212; doi:10.3390/polym13020257)
Supplement: Supplementary file 1 [file polymers-13-00257-s001.pdf]

polymers
MDPI

Supplementary Materials

3D-Printed Poly(ε-caprolactone)/Hydroxyapatite Scaffolds Modified with Alkaline Hydrolysis Enhance Osteogenesis In Vitro

Sangbae Park [1,†], Jae Eun Kim [1,†], Jinsub Han [2,3], Seung Jeong [1], Jae Woon Lim [1], Myung Chul Lee [1], Hyunmok Son [1], Hong Bae Kim [1], Yun-Hoon Choung [4], Hoon Seonwoo [5,*], Jong Hoon Chung [2,3,6,*] and Kyoung-Je Jang [7,8,*]

[1] Department of Biosystems & Biomaterials Science and Engineering, Seoul National University, Seoul 08826, Korea; sb92park@snu.ac.kr (S.P.); je6740@snu.ac.kr (J.E.K.); jsw3055@snu.ac.kr (S.J.); jwlim1130@snu.ac.kr (J.W.L.); josephmyungchul@gmail.com (M.C.L.); shmking@snu.ac.kr (H.S.); hbkim@snu.ac.kr (H.B.K.)
[2] Department of Biosystems Engineering, Seoul National University, Seoul 08826, Korea; rhineop@snu.ac.kr
[3] BK21 Global Smart Farm Educational Research Center, Seoul National University, Seoul 08826, Korea
[4] Department of Otolaryngology, Ajou University School of Medicine, Suwon 16499, Korea; yhc@ajou.ac.kr
[5] Department of Industrial Machinery Engineering, College of Life Sciences and Natural Resources, Sunchon National University, Suncheon 57922, Korea
[6] Research Institute of Agriculture and Life Sciences, Seoul National University, Seoul 08826, Korea
[7] Division of Agro-system Engineering, College of Agriculture and Life Science, Gyeongsang National University, Jinju 52828, Korea
[8] Institute of Agriculture & Life Science, Gyeongsang National University, Jinju 52828, Korea
† These two authors equally contributed to this study
* Correspondence: uhun906@gmail.com (H.S.); jchung@snu.ac.kr (J.H.C.); kj_jang@gnu.ac.kr (K.-J.J.); Tel.: +82-61-750-3261 (H.S.); +82-2-880-4601 (J.H.C.); +82-55-772-1898 (K.-J.J.)

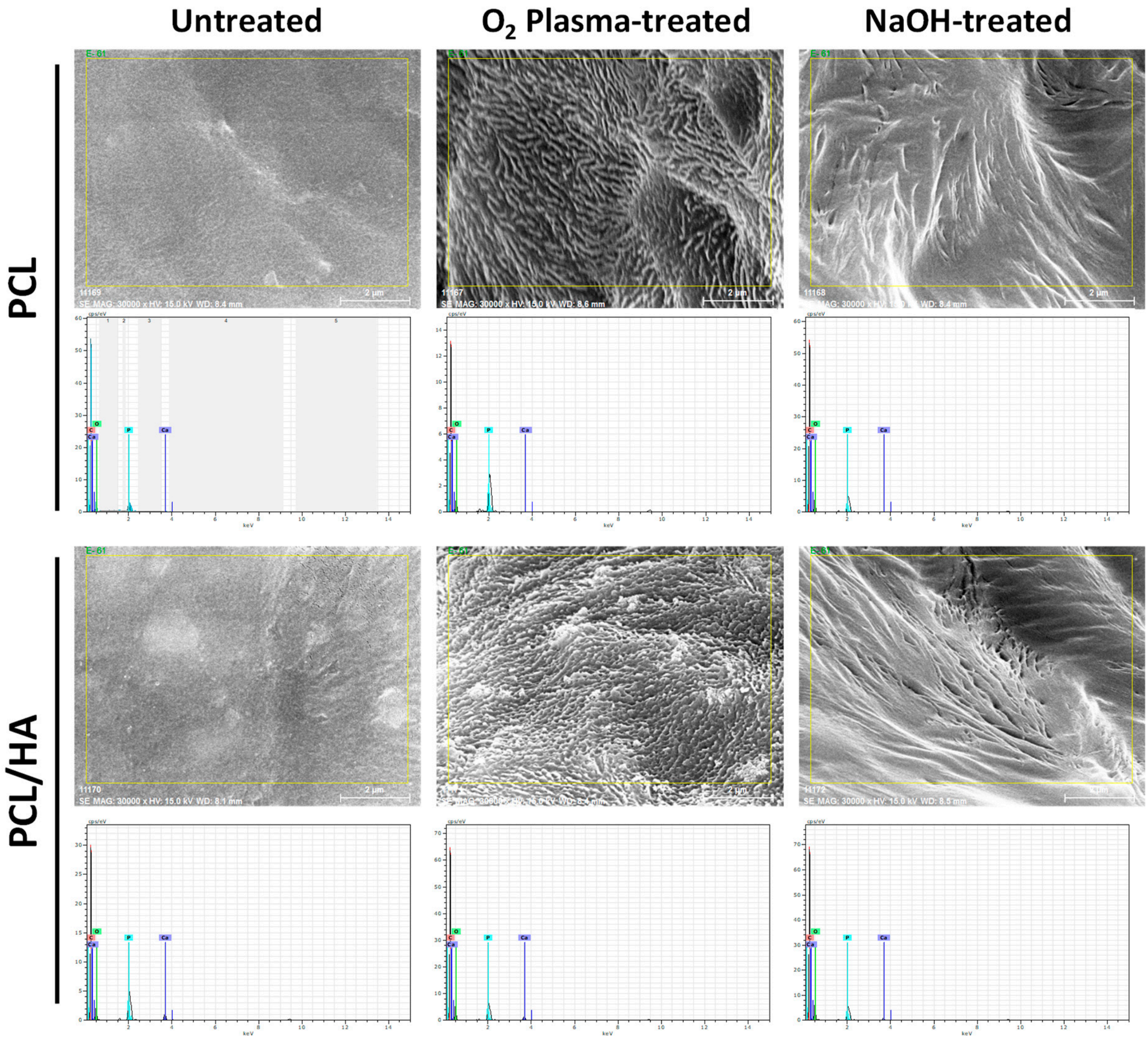

Figure S1. The EDS spectra of the PCL and PCL/HA scaffolds with different surface treatments.

Table S1. Elemental analysis of the scaffolds using EDS.

		C (wt. %)	O (wt. %)	Ca (wt. %)	P (wt. %)	Total (%)
PCL	Untreated	83.56	15.13	0.02	1.28	100
	O_2 Plasma-treated	80.76	14.62	0	4.61	100
	NaOH-treated	81.2	16.64	0	2.16	100
PCL/HA	Untreated	79.09	14.65	2.58	3.67	100
	O_2 Plasma-treated	78.61	17.20	1.72	2.47	100
	NaOH-treated	77.98	19.19	0.90	1.92	100

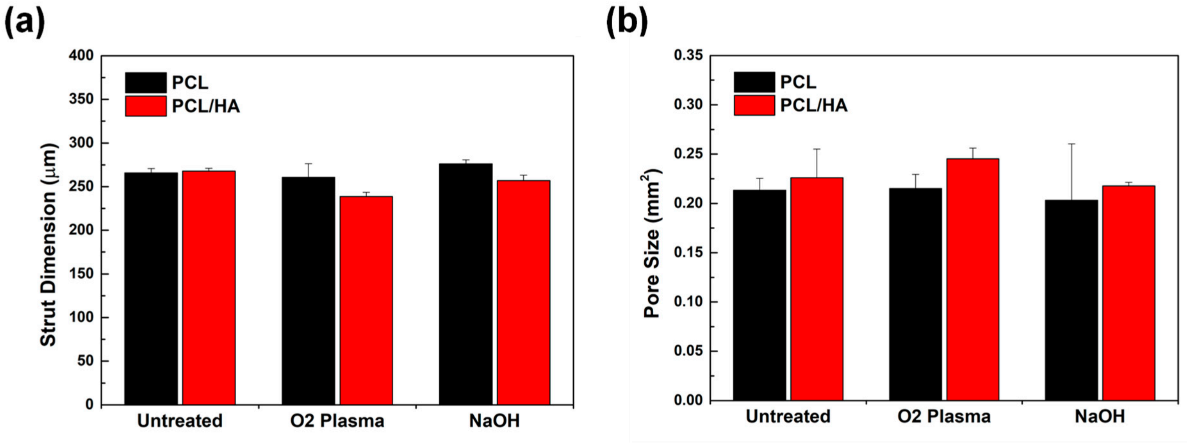

Figure S2. Strut and pore dimensions obtained for the different scaffolds.

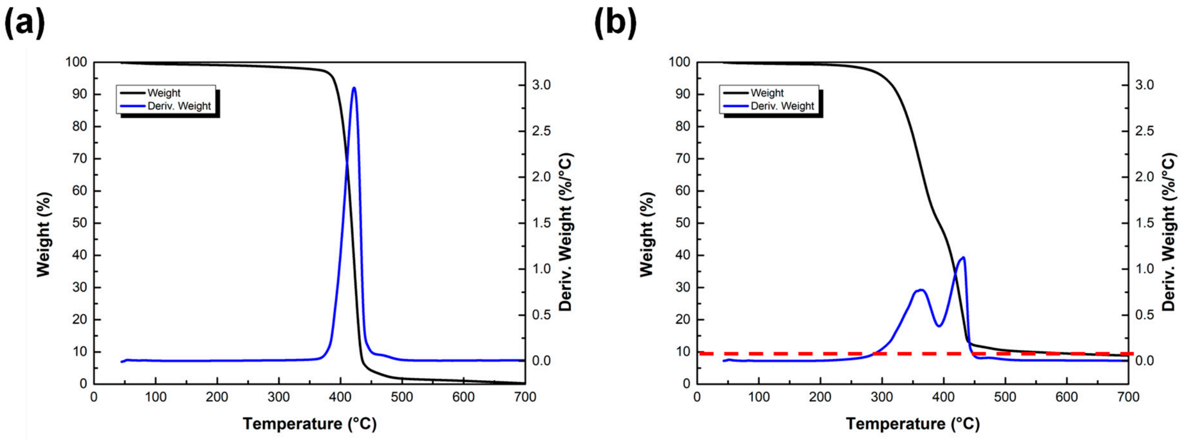

Figure S3. TGA curves of PCL and PCL/HA scaffolds.

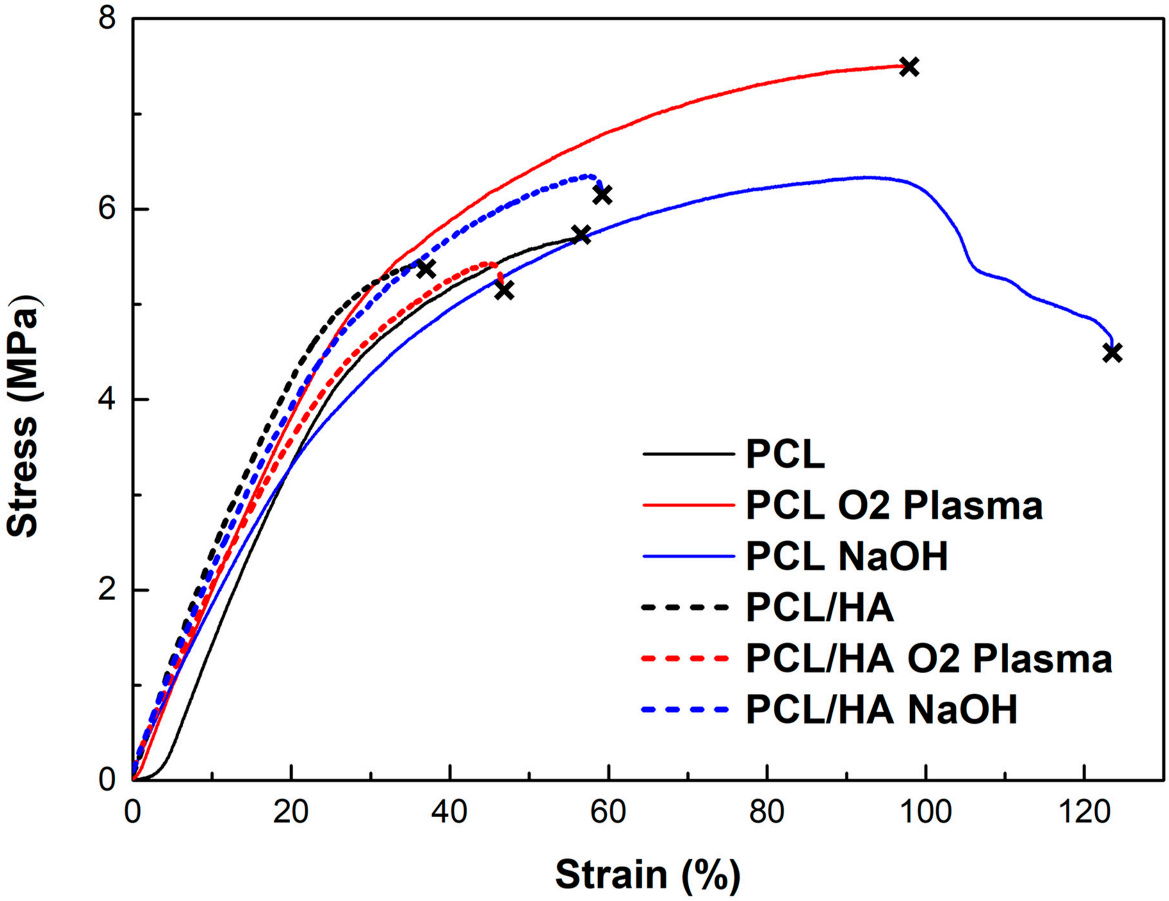

Figure S4. Stress-strain curves of PCL and PCL/HA scaffolds with different surface treatments. (×: Rupture Point, Black: untreated, Red: O_2 plasma-treated, and Blue: NaOH-treated).

Table S2. Mechanical properties of the scaffolds with different surface treatments.

		Elastic Modulus (Mpa)	Tensile Strength (Mpa)	Strain (%)
PCL	Untreated	18.25±3.14	5.87±0.52	106.51±9.51
	O_2 Plasma-treated	19.14±1.14	7.06±0.69	95.04±4.45
	NaOH-treated	18.38±2.28	6.97±0.63	144.51±7.01
PCL/HA	Untreated	19.69±2.13	4.97±1.39	32.38±4.01
	O_2 Plasma-treated	18.09±1.36	5.62±0.38	45.37±3.11
	NaOH-treated	19.01±0.98	6.12±0.41	54.24±2.27
